# Pathological evaluation of renal complications in children following allogeneic hematopoietic stem cell transplantation: a retrospective cohort study

**DOI:** 10.1186/s12887-023-03996-1

**Published:** 2023-04-21

**Authors:** Ru-Yue Chen, Xiao-Zhong LI, Qiang Lin, Han-Yun Tang, Ning-Xun Cui, Lu Jiang, Xiao-Mei Dai, Wei-Qing Chen, Fan Deng, Shao-Yan Hu, Xue-Ming Zhu

**Affiliations:** 1 Department of Nephrology and Immunology, Children’s Hospital of Soochow University, Suzhou, Jiangsu China; 2 Department of Hematology, Children’s Hospital of Soochow University, Suzhou, Jiangsu China; 3 Department of Pathology, Children’s Hospital of Soochow University, Suzhou, Jiangsu China

**Keywords:** Allogeneic hematopoietic stem cell transplantation (allo-HSCT), Renal pathology, Mesangial proliferative glomerulonephritis (MSPGN), Focal segmental glomerulosclerosis (FSGS), Membranoproliferative glomerulonephritis (MPGN), Thrombotic microangiopathy (TMA)

## Abstract

**Background:**

Allogeneic hematopoietic stem cell transplantation (allo-HSCT) is a curative therapy for hematologic malignancies and non-malignant disorders, such as aplastic anemia, fanconi anemia, and certain immune deficiencies. Post-transplantation kidney injury is a common complication and involves a wide spectrum of structural abnormalities, including glomerular (MSPGN, mesangial proliferative glomerulonephritis; FSGS, focal segmental glomerulosclerosis; MPGN, membranoproliferative glomerulonephritis; MCD, minimal change disease), vascular (TMA, thrombotic microangiopathy), and/or tubulointerstitial (TIN, tubulointerstitial nephritis; ATI, acute tubular injury). Renal biopsy is the gold-standard examination for defining multiple etiologies of kidney impairment. Although kidney injury following HSCT has been studied, little is known about the effects of allo-HSCT on renal pathology in pediatric patients.

**Methods:**

We retrospectively analyzed renal biopsy specimens from children with kidney injury after allo-HSCT and correlated results with clinical data in the last 10 years.

**Results:**

Among 25 children (18 males and 7 females), three patients had proteinuria indicating nephrotic syndrome (24-hour urinary total protein/weight > 50 mg/kg/d), nine patients had severely reduced estimated glomerular filtration rate (eGFR < 30 ml/min/1.73 m^2^) and four patients received kidney replacement therapy (KRT). The main pathologies identified from kidney biopsies were MSPGN (n = 12), FSGS (n = 12), MPGN (n = 5), TMA (n = 4), MCD (n = 3), diffuse glomerular fibrosis (DGF, n = 2), ATI and TIN, in isolation or combined with other pathologies. The median follow-up time was 16.5 (0.5 ~ 68.0) months. Three patients died of recurrent malignancy and/or severe infection, one child developed to end-stage renal disease (ESRD), six patients (24%) had elevated serum creatinine (SCr > 100µmol/l) and nine patients (36%) still had proteinuria.

**Conclusions:**

This study evaluates histomorphologic findings from kidney biopsies of pediatric recipients following allo-HSCT. Detailed evaluation of renal biopsy samples is helpful to elucidate the nature of renal insult, and may potentially identify treatable disease processes.

## Introduction

Advancements in hematopoietic stem cell transplantation (HSCT) have contributed greatly to improving the quality of life and extending the survival of patients with terminal diseases. Nevertheless, there have been increasing reports of kidney injury after HSCT. The incidence of acute kidney injury (AKI) after HSCT has been reported to vary as high as 10–75%, in which approximately 5% of patients required kidney replacement therapy (KRT) and 60% developed chronic kidney disease (CKD) [1, 2]. The causes are often multifactorial, and can include graft versus host disease (GVHD), effects of nephrotoxic medications, marrow infusion syndrome, hepatic sinusoidal obstruction syndrome, sepsis, and chronic infections (such as BK virus and adenovirus). The reported incidence of CKD after pediatric allogeneic hematopoietic stem cell transplantation (allo-HSCT) varies between 0% and 44%, and the main risk factor for CKD was found to be severe prolonged stage 2 or higher AKI, with an estimated glomerular filtration rate (eGFR) under 60 ml/min/1.73 m^2^ and a duration of 28 days or more [3]. Histopathological findings of kidney injury after HSCT mainly involve glomerular (MGN, membranous glomerulonephritis; MCD, minimal change disease; FSGS, focal segmental glomerulosclerosis), tubulointerstitial (TIN, tubulointerstitial nephritis; ATI, acute tubular injury; ATN, acute tubular injury necrosis) and vascular (TMA, thrombotic microangiopathy) [4, 5]. In this study, we discuss renal pathologic findings associated with kidney injury after allo-HSCT in pediatric patients, and determine associations with clinical factors.

## Methods

### Patients

We retrospectively analyzed the pathological and clinical data of children treated with allo-HSCT, diagnosed with post-transplantation kidney injury, and peformed renal biospy in the Department of Hematology, Pathology, Nephrology and Immunology, Children’s Hospital of Soochow University. Inclusion criteria: (1) receiving allo-HSCT treatment, including bone marrow (BM), peripheral blood stem cells (PB), and/or umbilical cord blood (UCB) from sibling, parents and/or unrelated donor for hematological malignancy or severe nonmalignant diseases; (2) complicating with renal injury after HSCT, including elevated serum creatinine (SCr) and decreased eGFR; (3) accepting percutaneous renal biopsies and histopathological examination, including light microscopy, immunofluorescence, and electron microscopy. Renal biopsy would be considered if proteinuria, and/or significant renal impairment defined by > 50% increase in serum creatinine from baseline level or eGFR < 60 ml/min/1.73 m^2^ on two occasions. Written informed consent to receive the renal biopsy and the collection of clinical and pathological data was obtained from all study participants including the parents or legal guardians of any participant under the age of 16. The study protocol was reviewed and approved by Children’s Hospital of Soochow University ethics committee. All methods were performed in accordance with the relevant guidelines and regulations.

### Treatment

All patients were treated with allo-HSCT including BM, PB, and/or UCB from sibling, parents and/or unrelated donor, in which case patients needed to receive GVHD prophylaxis (CSA, cyclosporine A; MTX, methotrexate; MMF, mycophenolate mofetil; TAC, tacrolimus; SRL, sirolimus; basiliximab). Before receiving an infusion of hematopoietic stem cells, the HSCT recipients were treated with a chemotherapeutic conditioning regimen including simustine (CCNU), busulfan (BU), cyclophosphamide (CY), cytosine arabinoside (Ara-C), fludarabine (FLU), anti-thymocyte globulin (ATG), cladribine (CDA), etoposide (VP16), decitabine (DAC), and/or rituximab. Pediatric patients received simultaneous treatment with anti-infection drugs, including antibiotics (penicillins, cephalosporins, macrolides and vancomycin), antiviral agents (aciclovir and ganciclovir), and/or antifungal drugs. (Table 1) After diagnosis of renal injury, glucocorticoid (approximately 1 mg/kg) and/or MMF (approximately 20 mg/kg) combined symptomatic treatment were applied in patients. CSA was reduced or discontinued. 4 children (patient 4, 7, 13, and 20) received KRT.

**Table 1:** Patient and hematopoietic stem cell transplantation parameters

Case	Age at HSCT (y)	Sex	Diagnosis	Donor	Cell source	Preparative regimen	GVHD prophylaxis	GVHD
1	10.9	M	AML	Sibling	BM + PB	CCUN + BU + CY + Ara-C + Rituximab + TBI	CSA + MTX	Intestinal and skin^2^
2	6	F	AA	Sibling	BM + PB	FLU + BU + CY + ATG	MMF + SRL	-
3	10.1	M	AA	Parents and unrelated^1^	BM + PB + UCB^1^	FLU + BU + CY + ATG + Rituximab	CSA + MMF + MTX	Skin^3^
4	11.5	M	ALL	Sibling	BM + PB	CCUN + BU + CY + Ara-C + TBI	CSA + MMF	Intestinal, skin and liver^3^
5	7	M	ALL	Parents and unrelated^1^	BM + PB + UCB^1^	CCUN + FLU + CY + Ara-C + ATG + TBI	CSA + MMF	Intestinal^3^
6	13.8	M	AA	Sibling	BM + PB	FLU + CY + ATG	CSA + MMF	Intestinal^3^
7	7.1	M	AA	Unrelated	PB	FLU + CY + ATG	CSA	Intestinal and skin^3^
8	9	F	MDS	Parents and unrelated^1^	BM + PB + UCB^1^	FLU + BU + CY + ATG	TAC + MMF	-
9	11.4	F	AA	Sibling and unrelated^1^	BM + PB + UCB^1^	FLU + BU + CY + ATG	TAC + MMF	-
10	4.4	M	WAS	Parents	BM + PB	FLU + BU + CY + ATG + Rituximab	CSA + MMF	Skin^2^
11	10.7	F	AA	Sibling	BM + PB	FLU + CY + ATG	TAC + MTX	-
12	10.6	F	AA	Sibling	BM + PB	FLU + CY + ATG	CSA + MTX	-
13	14.6	F	AML	Sibling	PB	NA	TAC + MMF	-
14	7.8	M	ALL	Parents	BM + PB	CCUN + FLU + BU + CY + Ara-C + ATG + TBI	CSA + MMF + MTX	-
15	11.3	M	AA	Parents and unrelated^1^	BM + PB + UCB^1^	FLU + BU + CY + ATG + Rituximab	TAC + MMF + MTX	-
16	1	M	WAS	Sibling	PB	FLU + BU + CY + ATG	CSA + MMF	-
17	7	M	FA	Sibling	BM + PB	FLU + BU + CY + ATG	TAC + MMF + MTX	-
18	15.6	M	ALL	Unrelated	UCB	CCUN + FLU + BU + CY + Ara-C + TBI	CSA + MMF	Skin^3^
19	13.9	M	AML	Unrelated	PB	CDA + Bu + CY + Ara-C + ATG + TBI	TAC + MMF	-
20	13.7	M	CAEBV	Parents and unrelated^1^	BM + PB + UCB^1^	FLU + BU + VP-16 + ATG + Rituximab	CSA + MMF + MTX	Intestinal and skin^2^
21	2.4	M	WAS	Unrelated	UCB	FLU + BU + CY + ATG	CSA + MMF	-
22	8.6	M	AA	Parents and unrelated^1^	BM + PB + UCB^1^	FLU + BU + CY + ATG + Rituximab	TAC + MMF + MTX	Intestinal and skin^3^
23	5	M	AML	Unrelated	UCB	DAC + Bu + CY + Ara-C + ATG + TBI	CSA + MMF	Intestinal and skin^3^
24	13.1	F	AA	Parents	BM + PB	FLU + BU + CY + ATG + Rituximab	TAC + MMF + MTX	Intestinal and skin^3^
25	3.2	M	AML	Parents and unrelated^1^	BM + PB + UCB^1^	CCUN + BU + CY + Ara-C + TBI	CSA + MMF + MTX + Basiliximab	Intestinal and skin^2^

^1^ Umbilical cord blood originated from unrelated donor and bone marrow and peripheral blood stem cells originated from sibling or parents

^2^ cGVHD (chronic graft versus host disease)

^3^ aGVHD (acute graft versus host disease)

HSCT, hematopoietic stem cell transplantation; AML, acute myelogenous leukemia; AA, aplastic anemia; ALL, acute lymphocytic leukemia; MDS, myelodysplastic syndrome; WAS, Wiskott-Aldrich syndrome; FA, Fanconi anemia; CAEBV, chronic active Epstein-Barr virus infection; BM, bone marrow; PB, peripheral blood stem cells; UCB, umbilical cord blood; CCNU, simustine; BU, busulfan; CY, cyclophosphamide; Ara-C, cytosine arabinoside; FLU, fludarabine; ATG, anti-thymocyte globulin; TBI, total body irradiation; CDA, cladribine; VP16, etoposide; DAC, decitabine; CSA, cyclosporine A; MTX, methotrexate; MMF, mycophenolate mofetil; SRL, sirolimus; TAC, tacrolimus

### Monitoring of kidney function

Renal dysfunction was defined as elevated SCr and decreased eGFR. Clinical and laboratory evaluations were performed to assess renal function, including age (years), height (cm), weight (kg), 24-hour urinary total protein (24U-TP, mg/d), urinary protein (UP, mg/dl), serum creatinine (SCr, umol/L) and serum albumin (ALB, g/L). We used the updated Schwartz formula [(K × height)/SCr] with the modification of K = 36.5 (girls and boys aged 0–12 years) or K = 40 (boys aged 12–18 years) for the calculation of eGFR in patients aged < 18 years, as previously described [3]. (Table 2)

**Table 2:** Kidney function parameters

Case	The time between initial diagnosis and HSCT (m)	Renal injury before HSCT/eGFR	Renal injury at HSCT/eGFR	The time between renal injury and HSCT (m)	The time between renal biopsy and HSCT (m)	At renal biopsy				Follow-up		
						SCr	eGFR	ALB	24U-TP/Wt	Months	SCr	UP
1	4.7	—	—	5.7	14.2	197.0	30	40.2	NA	50.8	151.7	+
					47.2	117.6	56	39.5	90.8			
2	2.3	—	—	3.6	4.8	62.7	70	46.9	NA	0.8	59.7	+
					62.7	71.1	77	38.9	45.6			
3	35	112	67	—	9.3	180.2	29	44.7	17.6	2.3	Die	Die
					21.9	146.8	36	39.8	NA			
4	7.3	—	—	2.3	2.5	306.1	19	43.6	0.3	68.0	52.8	—
5	7.8	—	—	2.5	7.7	105.0	44	36.9	NA	1.7	Die	Die
6	1.3	—	—	1.7	3.9	268.0	26	39.3	27.5	8.7	82.6	+
7	40.4	43	41	—	1.9	260.0	19	43.9	NA	0.5	ESRD	ESRD
8	23.4	—	—	0.4	1.7	95.0	56	51.2	NA	20.4	54.2	—
9	72.5	—	—	0.8	2.7	67.0	90	40.1	1.8	12.7	40.4	+
10	50.6	—	—	3.5	9.7	176.2	22	56.6	NA	19.0	177.5	—
11	12.9	—	—	1.7	4.7	47.0	113	43.0	12.1	14.8	102.5	—
12	5.4	—	—	1.5	2	90.0	58	44.2	NA	42.8	60.8	—
13	3.9	—	—	1.1	1.6	232.2	27	42.8	2.2	37.5	65.5	—
14	70.4	—	—	1.3	1.6	84.1	59	45.7	53.5	28.4	52.2	—
15	106.4	78	86	—	7.5	126.0	44	46.6	1.8	15.1	59.0	—
16	10.7	—	—	21.9	34.8	106.7	34	45.6	14.4	26.4	169.8	+
17	4.3	—	—	7.0	7.1	362.0	12	42.3	7.9	16.5	44.5	—
18	5.6	—	—	11.7	15.9	106.0	63	45.1	8.5	24.1	95.0	+
19	4	—	—	0.8	2.4	78.0	91	43.7	1.3	25.2	68.6	—
20	9.8	—	—	7.8	9.7	102.0	59	39.3	7.0	11.2	Die	Die
21	28.7	—	—	37.6	54.9	74.8	57	40.8	6.4	24.9	70.8	+
22	23.8	51	98	—	11	111.3	45	41.0	1.7	19.8	203.3	+
23	16.8	—	—	6.9	11.8	127.1	32	38.0	NA	16.4	78.5	—
24	3.8	—	—	5.7	8.8	301.2	19	42.1	60.0	2.7	47.7	+
25	3.4	—	—	8.6	11.1	222.3	16	33.1	13.0	1.2	281.3	—

HSCT, hematopoietic stem cell transplantation; SCr, serum creatinine; eGFR, estimated glomerular filtration rate; ALB, serum albumin; 24U-TP/Wt, 24-hour urinary total protein/weight; UP, urinary protein; ESRD, end stage renal disease

### Evaluation of renal biopsy samples

Renal biopsy data were available for all patients. Histopathologic findings including light microscopy (HE, PAS, PASM and Masson staining) and immunofluorescence (IgA, IgG, IgM, C3, C1q, α3 chains, α5 chains, and fibrinogen) were evaluated by renal pathologists using standard criteria in the Department of Pathology, Children’s Hospital of Soochow University, which included evaluation of glomeruli, tubules/interstitium, and vessels. Tissue for electron microscopy analysis was processed by Shanghai Navy Medical Institute or Nanjing KingMed for clinical laboratory assessment.

## Results

### Patients and hematopoietic stem cell transplantation

There were 1198 children underwent allo-HSCT between 2012 and 2022. A cohort of 25 children (18 males and 7 females) met the inclusion criteria and was enrolled. The median age at allo-HSCT was 10.1 (1 ~ 15.6) years. The indications for transplantation included aplastic anemia (AA, n = 10), acute myelogenous leukemia (AML, n = 5), acute lymphocytic leukemia (ALL, n = 4), Wiskott-Aldrich syndrome (WAS, n = 3), myelodysplastic syndrome (MDS, n = 1), Fanconi anemia (FA, n = 1), and chronic active Epstein-Barr virus infection (CAEBV, n = 1). The median time between initial diagnosis and HSCT was 9.8 (1.3 ~ 106.4) months. Following allo-HSCT, 13 of these 25 pediatric patients exhibited evidence of GVHD, including 9 acute GVHD (aGVHD) and 4 chronic GVHD (cGVHD), which mainly involved intestinal, skin, and/or liver (Table 1). The majority of patients diagnosed with GVHD refer to clinical evidence of GVHD. Only two patients with intestinal GVHD received enteroscopy.

### Kidney injury

Four children in this cohort had renal injury before HSCT, which mainly manifested as elevated SCr and reduced eGFR. The remaining 21 patients had renal injury after transplantation, and the median time from initial renal injury to HSCT was 3.5 (0.4 ~ 37.6) months. The mean level of SCr was 150.8 (47.0 ~ 362.0) µmol/L and 24U-TP/Wt was 19.7 (0.3 ~ 90.8) mg/kg/d at time of renal biopsy. The serum albumin levels were within the normal range. Among the 25 patients, 3 children (patient 1, 14, and 24) had a large amount of proteinuria, indicating potential nephrotic syndrome (24U-TP/Wt > 50 mg/kg/d), 9 patients had severely reduced eGFR (eGFR < 30 ml/min/1.73 m^2^) and 4 children (patient 4, 7, 13, and 20) received KRT (Table 2).

### Renal pathology

**Fig. 1:**
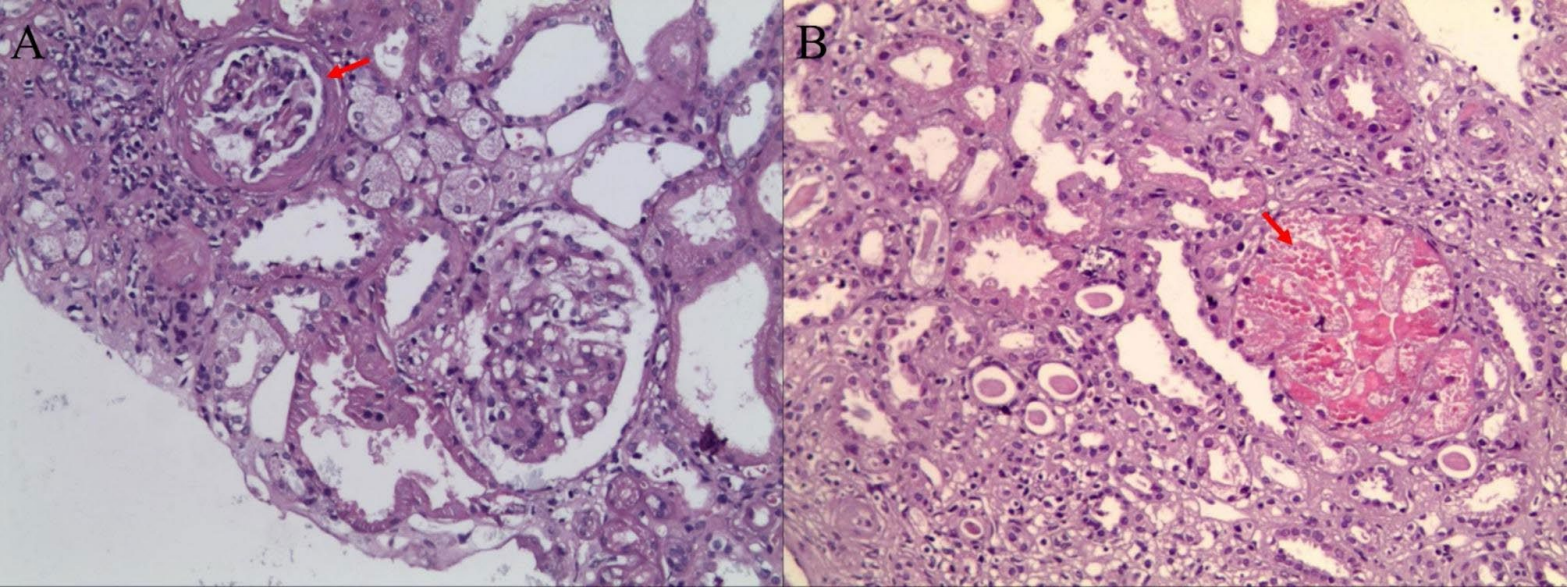
**A**. Example of FSGS (patient 3, PAS×200), including focal glomerular cystic wall fibrous hyperplasia and thickening. **B**. Example of TMA (patient 7, HE×200), including vascular wall thickening and necrosis, lumen occlusion, and microthrombosis

A total of 28 renal biopsies from 25 pediatric patients who had previously undergone allo-HSCT were identified; 3 patients received renal biopsy twice. The median time between renal biopsy and HSCT was 8.3 (1.6 ~ 62.7) months and the median time from initial renal injury to biopsy was 3.1 (0.1 ~ 59.1) months. The pathological findings of the kidney biopsies included Mesangial proliferative glomerulonephritis (MSPGN, n = 12), FSGS (n = 12), Membranoproliferative glomerulonephritis (MPGN, n = 5), TMA (n = 4), MCD (n = 3), diffuse glomerular fibrosis (DGF, n = 2), and ATI and TIN, which were in isolation or combined with other pathologies. Eight patients demonstrating MSPGN had evidence of GVHD. Both the median time from renal biopsy to HSCT and median time from initial renal injury to biopsy in children with MSPGN were shorter than in FSGS. Evidences of FSGS were found in 12 renal biopsies from 11 patients, which were characterized as segmental sclerosis in the glomeruli and were mostly accompanied by ATI or TIN. Two of the FSGS cases were combined with MPGN, and 2 cases were complicated with TMA. Seven patients with FSGS had evidence of GVHD. Five renal biopsy specimens from 4 patients (patient 1, 2, 11, and 16) demonstrated MPGN, in which 2 were combined with DGF and TIN, 1 was complicated with FSGS and TIN, 1 was complicated with TMA and ATI, and 1 was complicated with FSGS, TMA and TIN. Both the median time from renal biopsy to HSCT and the median time from initial renal injury to biopsy were longer compared with MSPGN, FSGS, and MCD. MCD was seen in 3 children (patient 12, 13, and 14), presented as podocyte fusion by electron microscopy and was negative upon immunofluorescent assessment. One patient exhibited a large amount of proteinuria at the level of nephrotic syndrome. Of the 3 patients with MCD, the median time between initial renal injury and biopsy was 0.5 (0.3 ~ 0.5) months and the median time from allo-HSCT to kidney biopsy was 1.6 (1.6 ~ 2.0) months, which were shorter compared with other pathological types. Two specimens from secondary renal biopsies in patient 1 and 2 exhibited DGF, and the time from renal biopsy to allo-HSCT was 47.2 and 62.7 months, respectively. TMA was seen in 4 children (patient 2, 7, 11, and 16), and the median time from renal biopsy to HSCT and from initial renal injury to biopsy was 4.75 (1.9 ~ 34.8) and 2.45 (1.2 ~ 12.9) months, respectively. Four children (patient 3, 7, 15, and 22) had renal injury before and during HSCT, and the renal pathology of these four patients showed FSGS and TIN; of these, patient 7 was complicated with TMA and received KRT. The lesions in renal tubules and interstitium mainly included ATI (n = 4) and TIN (n = 19), most of which were associated with MSPGN, FSGS, MPGN, TMA, or DGF. Only two patients demonstrated TIN in isolation. The histopathological manifestations of ATI involved vacuolar degeneration in tubular epithelial cells and granular, hyaline, or protein casts in the tubular lumen. The pathological lesions marked TIN mainly included tubulointerstitial inflammatory cell infiltration and/or fibrosis. Both the median time from renal biopsy to HSCT and the median time from initial renal injury to biopsy in children with ATI were shorter than in TIN (Tables 3 and 4; Fig. 1).

**Table 3:** Renal pathology determined by light microscopy

Case	Diagnosis	Light microscopy						
		Glomerulus		Tubules		Interstitium		Vessels
		Mesangial proliferation	Fibrosis	Atrophy	Granular, hyaline or protein cast	Inflammatory infiltration	Fibrosis	
1	FSGS, MPGN, TIN	Severe	1/27	+	-	+	-	-
1	DGF, MPGN, TIN	Moderate-severe	12/20	-	+	+	+	-
2	TMA, ATI	-	-	+	+	-	-	Microthrombosis
2	DGF, MPGN, TIN	Moderate-severe	9/14	-	-	+	+	-
3	FSGS, TIN	-	3/9	+	+	+	+	-
3	FSGS, TIN	-	1/6	-	-	+	+	-
4	FSGS, MSPGN, ATI	Mild	5/19	-	-	-	+	-
5	MSPGN, TIN	Severe	-	-	-	+	+	-
6	FSGS, MSPGN	Mild	1/11	-	-	-	-	-
7	FSGS, TMA, TIN	-	1/15	-	+	+	+	Microthrombosis
8	FSGS, MSPGN, ATI	Mild	1/13	-	+	-	-	-
9	TIN	-	-	-	-	+	-	-
10	TIN	-	-	-	+	+	+	-
11	MPGN, TMA, ATI	Moderate	-	-	+	-	-	Microthrombosis
12	MCD	-	-	-	-	-	-	-
13	MCD	-	-	+	+	-	-	-
14	MCD	-	-	-	-	-	-	-
15	FSGS, TIN	-	4/16	-	-	+	-	-
16	FSGS, MPGN, TMA, TIN	Moderate-severe	23/56	+	-	+	+	Thickening, Microthrombosis
17	MSPGN, TIN	Mild	-	+	+	+	+	-
18	FSGS, TIN	-	1/8	+	+	+	+	-
19	MSPGN	Mild	-	+	-	-	-	-
20	MSPGN, TIN	Mild	-	-	+	+	+	-
21	FSGS, MSPGN, TIN	Moderate-severe	4/20	-	+	+	-	-
22	FSGS, MSPGN, TIN	Mild	4/17	-	+	+	+	-
23	MSPGN, TIN	Mild-moderate	-	+	+	+	+	-
24	MSPGN, TIN	Mild	-	+	-	+	+	-
25	MSPGN, TIN	Mild	-	+	+	+	+	-

MPGN, membranoproliferative glomerulonephritis; MSPGN, mesangial proliferative glomerulonephritis; MCD, minimal change disease; TIN, tubulointerstitial nephritis; DGF, diffuse glomerular fibrosis; TMA, thrombotic microangiopathy; FSGS, focal segmental glomerulosclerosis; ATI, acute tubular injury

**Table 4:** Renal pathology determined by immunofluorescence and electron microscopy

Case	Immunofluorescence								Electron microscopy
	IgG	IgM	IgA	C1q	C3	Fibrinogen	ɑ3	ɑ5	
1	±/G	—	—	—	—	—	NA	NA	Podocyte fluff; interstitial lymphocyte infiltration; partial vascular loop degeneration
1	—	+/G	—	+/G	+/G	—	—	—	Glomerulosclerosis; tubular atrophy; interstitial fibrosis, lymphocyte and monocyte infiltration
2	—	2+/G	—	—	—	+/G	—	—	RBCs gathered in capillaries lumen and arterioles wall thickening; TECs vacuolar degeneration
2	—	1+/G	1+/G	—	1+/T	—	—	—	EDD; GBM thickening and delamination; interstitial inflammatory infiltration
3	—	—	—	—	—	—	—	—	GBM shrinking; interstitial inflammatory infiltration
3	—	—	—	—	—	—	—	—	GBM laceration; interstitial fibrosis and inflammatory infiltration
4	—	—	—	—	1+/I	—	NA	NA	Podocyte fluff; TECs vacuolar degeneration
5	—	—	—	—	1+/I	—	NA	NA	Interstitial fibrosis and lymphocyte infiltration; vascular loop occlusion
6	—	—	—	—	1+/I	1+/G	NA	NA	Mesangial proliferation
7	—	1+/I, G	1+/T	1+/I	1+/I	—	NA	NA	Podocyte fluff; vascular loop degeneration
8	1+/T	—	1+/T	—	—	—	—	—	NA
9	—	—	—	—	1+/T	—	—	—	GBM thickening; TECs vacuolar degeneration; interstitial inflammatory infiltration
10	±/G	±/G	—	—	1+/T	1+/T	—	—	GBM ischemia and shrinking; EDD; TECs vacuolar degeneration; tubular atrophy; interstitial inflammatory infiltration
11	—	—	—	—	1+/I	—	—	—	Podocyte fusion; RBCs gathered in capillaries lumen and arterioles wall thickening
12	—	—	—	—	—	—	—	—	Podocyte fusion
13	—	—	—	—	—	—	—	—	Podocyte fusion
14	—	—	—	—	—	—	—	—	Podocyte fusion
15	1+/G	—	—	—	—	—	—	—	GBM shrinking; TECs vacuolar degeneration
16	—	—	1+/T	1+/G	1+/G	—	—	—	EDD; focal segmental glomerulosclerosis
17	—	—	—	—	—	—	—	—	GBM shrinking; TECs vacuolar degeneration; interstitial inflammatory infiltration
18	—	—	—	—	—	—	—	—	GBM shrinking; TECs vacuolar degeneration; tubular atrophy; interstitial inflammatory infiltration
19	—	2+/G	±/G	—	1+/I	—	—	—	EDD; TECs vacuolar degeneration
20	—	1+/G	—	—	1+/I, T	—	—	—	GBM shrinking
21	—	—	1+/G	—	—	—	—	—	Tubular and interstitial inflammatory infiltration
22	—	1+/T	—	—	—	—	—	—	EDD; TECs vacuolar degeneration; interstitial inflammatory infiltration
23	—	—	—	—	—	—	—	—	EDD; TECs vacuolar degeneration; tubular atrophy
24	—	—	—	—	—	—	—	—	EDD; TECs vacuolar degeneration; tubular atrophy; interstitial inflammatory infiltration
25	—	—	—	—	—	—	—	—	TECs vacuolar degeneration; interstitial inflammatory infiltration

G, Immunofluorescence glomerular capillary loop and/or mesangial staining; T, Immunofluorescence renal tubule basement membrane and/or epithelial cells staining; I, Immunofluorescence renal interstitium and vascular wall staining; NA, not available; RBCs, red blood cells; TECs, tubular epithelial cells; EDD, electron dense deposits; GBM, glomerular basement membrane

### Prognosis

Patient median follow-up time was 16.5 (0.5 ~ 68.0) months. Three patients died of recurrent malignancy and/or severe infection, one child developed end-stage renal disease (ESRD) and was lost to follow-up, six patients (24%) had elevated SCr (> 100 µmol/l), and nine patients (36%) had persistent proteinuria ( + ~ 3+). Excluding combination with FSGS and/or TIN, only one patient demonstrated MSPGN in isolation; this patient had a relatively good prognosis, including normal SCr levels and negative proteinuria at follow-up of 25.2 months. In the 11 patients with FSGS, six (55%) had elevated SCr (> 100 µmol/l) and/or proteinuria, one progressed to ESRD, and one died during follow-up (0.5 ~ 68.0 months, mean 23 months). All four children (patient 1, 2, 11, and 16) who demonstrated MPGN had elevated SCr (> 100 µmol/l) and/or proteinuria at follow-up (0.8 ~ 50.8 months, mean 23.2 months), indicating a relatively poor prognosis. Three children (patient 12, 13, and 14) with MCD had SCr levels of 52.2 ~ 65.5, and negative proteinuria at follow-up (28.4 ~ 42.8 months, mean 36.2 months), indicating a relatively good prognosis. The children with TMA had relatively poor prognosis; which patient 2 with TMA and ATI underwent a second renal biopsy after nearly 5 years and demonstrated DGF, MPGN and TIN; patient 7 with TMA, FSGS, and TIN received KRT and progressed to ESRD; patient 11 and 16 continued to exhibit elevated SCr (> 100 µmol/l) and/or proteinuria at follow-up (14.8 and 26.4 months, respectively). Four children (patient 3, 7, 15, and 22) who had renal injury before and during HSCT had a relatively poor prognosis; patient 3 died of recurrent malignancy, patient 7 progressed to ESRD and was lost to follow-up, and patient 22 had elevated SCr (> 200 µmol/l) and proteinuria (+) at follow-up of 19.8 months. Patient 15 had normal SCr levels and negative proteinuria at follow-up of 15.1 months (Table 2).

## Discussion

Hematopoietic stem cell transplantation (HSCT) is a proven treatment for hematopoietic malignancies, some solid tumors, and other marrow or immune disorders. The kidney is exposed to a large variety of injurious insults before, during, and after HSCT, leading to a high incidence of AKI and CKD [1–3]. Post-transplantation renal injury may be related to a combination of factors including chemotherapy, radiation, infection, immunosuppressive agents, and GVHD [1]. Kidney biopsies can reveal abnormalities in glomeruli, tubules, interstitium, and vessels, which are useful for confirming risk factors and defining underlying pathological mechanisms to guide therapy. In this retrospective study, we reviewed the renal pathology of a cohort of pediatric allo-HSCT recipients combined with clinic data.

At present, MGN is the most common glomerular lesion in the setting of HSCT, followed by MCD. Brukamp, et al. [6] reported that MGN accounts for almost two-thirds of nephrotic syndrome after HSCT, followed by MCD in nearly one quarter of patients. Moreover, the literature revealed a close temporal connection between the development of nephrotic syndrome shortly after stopping immunosuppression and diagnosing GVHD, which was considered glomerular lesions after HSCT may represent the renal manifestation of GVHD [6]. However, in our pediatric study, 43% (12/28) of renal specimens showed MSPGN and 18% (5/28) demonstrated MPGN, which differs from the previous reports. In this pediatric cohort, only 3 patients had MCD and 1 of them exhibited a large amount of proteinuria consistent with nephrotic syndrome. Eight patients with MSPGN had evidence of GVHD. GVHD is primarily attributed to an imbalance of T cells, wherein alloreactive donor T cells responding to host histocompatibility antigens, whereas some reports demonstrate that rituximab is efficacious in the treatment of cGVHD and MGN [4, 7]. Approximately 60–70% of patients with nephrotic syndrome achieve complete resolution after treatment with immunosuppressive regimens, in which the complete resolution rate in MCD was higher than in MGN [7, 8]. In our study, three children with MCD had normal levels of SCr and negative proteinuria in follow-up, suggesting a relatively good prognosis. Excluding cases combined with FSGS and/or TIN, only one patient demonstrated MSPGN in isolation; this patient had a relatively good prognosis with normal SCr levels and negative proteinuria. However, all four children with MPGN had elevated SCr and/or proteinuria, indicating a relatively poor prognosis. Rikako Hiramatsu, et al. [9] reported that HSCT-related MGN developed in 5 patients only after using UCB transplantation, but did not report the development of MGN after unrelated BM transplantation; this was considered to be related to the presence of HLA antibodies against UCB units as a causative factor of MGN.

FSGS after HSCT is reported in a minority of cases, generally presents with nephrotic syndrome, and can be explained by the immunological damage incurred during chronic GVHD progression [10–13]. However, the overall incidence of FSGS that we observed in this cohort (12/28) was higher than what has been reported by others in allo-HSCT, which may be associated with the elevated SCr levels prior to transplantation. Previous literature showed patients who develop acute kidney injury early after transplantation are at increased risk of progression to chronic kidney failure later in the post-transplantation course [14, 15]. The renal pathology of all four cases who had renal dysfunction prior to HSCT showed FSGS and TIN, which suggested pre-transplantation renal injure was the main risks for the CKD after allo-HSCT. In the 11 patients with FSGS, 6 (55%) of them had significantly elevated SCr and/or proteinuria, one of them progressed to ESRD, and one patient died during follow-up, suggesting a relatively poor prognosis. Seven patients with FSGS had evidence of GVHD, which is consistent with the speculation in previous literature that FSGS is related to GVHD [10]. Meanwhile, both the median time from renal biopsy to HSCT and the median time from initial renal injury to biopsy in children with FSGS were longer than in MSPGN, TMA, and MCD.

TMA is a severe complication in HSCT recipients, and the kidney is most commonly affected by vascular endothelial cell injury, resulting in renal dysfunction, proteinuria and hypertension [16]. Eleni Gavriilaki, et al. [17] reported that 15.5% of HSCT patients were diagnosed with transplant-associated thrombotic microangiopathy (TA-TMA) and total body irradiation, viral infections, and GVHD remained independent predictors of TA-TMA. Meanwhile, TA-TMA has a high mortality rate and increases the risk for CKD after HSCT [16, 18, 19]. The histopathologic feature of HSCT-TMA included vascular endothelial cell injury, resulting in microangiopathic hemolytic anemia, platelet consumption, fibrin deposition in the microcirculation, and tissue damage, finally leading to the loss of integrity of the glomerular filtration barrier, which is similar with hemolytic uremic syndrome (HUS) and thrombotic thrombocytopenic purpura (TTP) [20]. Both clinical data and murine experiment demonstrated proposed mechanism containing complement activation and endothelial variant of GVHD [20–22]. Elevated lactate dehydrogenase, proteinuria, and hypertension were considered as the earliest markers of TMA, and proteinuria and elevated markers of complement activation at TMA diagnosis are associated with poor outcome [18]. Many risk factors including aGVHD (especially grade 2–4), unrelated donor transplants and exposure to calcineurin inhibitors (CNIs) were considered to be related to the development of HSCT-TMA [21]. In our patients, four were complicated with TMA and had a relatively poor prognosis; of these, one patient with TMA and ATI underwent a second renal biopsy after nearly 5 years and demonstrated DGF, MPGN, and TIN; one patient with TMA, FSGS, and TIN received KRT and progressed to ESRD; two patients had persistent elevated SCr and/or proteinuria at follow-up. Approximately 50–63% of patients with TA-TMA respond to withdrawal of the offending agent (CNIs) and therapeutic plasma exchange (TPE) [23]. Eculizumab is a humanized monoclonal immunoglobulin G antibody binding to complement protein C5 and preventing complement-mediated TMA in patients, which is approved by the Food and Drug Administration for the treatment of paroxysmal nocturnal hemoglobinuria and atypical HUS. Sonata Jodele, et al. [24] reported the experience of 64 pediatric HSCT recipients with high risk TA-TMA and multi-organ injury treated with the complement blocker eculizumab and demonstrated significant improvement at one year post-HSCT survival. The anti-CD20 monoclonal antibody rituximab has been reported to have response without notable treatment-related toxicities [20, 23]. Other pharmacologic treatment options include defibrotide, vincristine and pravastatin in cases of TMA [23].

Compared with glomerular disease and TMA, tubulointerstitial lesions associated with HSCT are less commonly reported. Typical histologic features of acute interstitial nephritis (ATN) include ectatic tubules lined by flattened epithelial cells exhibiting loss of brush border and reactive nuclei, interstitial inflammatory cell infiltrate, considered to be a manifestation of drug hypersensitivity, postviral syndrome, and inflammatory or regenerative response to tubular injury [8]. In a review of the literature, Troxell ML, et al. [8] showed that over half of the specimens in renal lesions of HSCT patients demonstrated substantial interstitial fibrosis and tubular atrophy, and over half showed global glomerulosclerosis. El-Seisi S. et al. [25] reported that tubulitis and interstitial fibrosis were observed in 67% and 62% of autopsy of patients who died after HSCT, respectively. In our study, the lesions in renal tubules and interstitium mainly included ATI (4/28) and TIN (19/28), most of which were combined with MSPGN, FSGS, MPGN, TMA or DGF. Only two cases demonstrated TIN in isolation. Both the median time from renal biopsy to HSCT and the median time from initial renal injury to biopsy in children with ATI were shorter than patients with TIN.

## Conclusions

Morphologic renal lesions after allo-HSCT are often indicative of multiple pathologies, with glomerular, tubulointerstitial, and/or vascular lesions coexisting. Multiple pathologies are frequently seen, and correlation with clinical history including primary disease, preparative regimen, immunosuppressive treatment, and GVHD is important. At present, this is the first study of pediatric renal pathology after allo-HSCT we have known, which is in contrast to previous studies on non-pediatric HSCT recipients. Kidney biopsies are needed to confirm risk factors and to better define the underlying mechanisms of renal insult following HSCT in order to improve therapies to prevent these complications.

## Data Availability

The datasets used and/or analysed during the current study available from the corresponding author on reasonable request.

## Abbreviations

Allo-HSCT: Allogeneic hematopoietic stem cell transplantation
MSPGN: Mesangial proliferative glomerulonephritis
FSGS: Focal segmental glomerulosclerosis
MPGN: Membranoproliferative glomerulonephritis
MCD: Minimal change disease
TMA: Thrombotic microangiopathy
TIN: Tubulointerstitial nephritis
ATI: Acute tubular injury
eGFR: Estimated glomerular filtration rate
KRT: Kidney replacement therapy
DGF: Diffuse glomerular fibrosis
ESRD: End-stage renal disease
SCr: Serum creatinine
AKI: Acute kidney injury
CKD: Chronic kidney disease
GVHD: Graft versus host disease
MGN: Membranous glomerulonephritis
BM: Bone marrow
PB: Peripheral blood stem cells
UCB: Umbilical cord blood
CSA: Cyclosporine A
MTX: Methotrexate
MMF: Mycophenolate mofetil
TAC: Tacrolimus
SRL: Sirolimus
CCNU: Simustine
BU: Busulfan
CY: Cyclophosphamide
Ara-C: Cytosine arabinoside
FLU: Fludarabine
ATG: Anti-thymocyte globulin
CDA: Cladribine
VP16: Etoposide
DAC: Decitabine
24U-TP: 24-hour urinary total protein
UP: Urinary protein
ALB: Serum albumin
AA: Aplastic anemia
AML: Acute myelogenous leukemia
ALL: Acute lymphocytic leukemia
WAS: Wiskott-Aldrich syndrome
MDS: Myelodysplastic syndrome
FA: Fanconi anemia
CAEBV: Chronic active Epstein-Barr virus infection
aGVHD: Acute graft-versus-host disease
cGVHD: Chronic graft-versus-host disease
TA-TMA: Transplant-associated thrombotic microangiopathy
HUS: Hemolytic uremic syndrome
TTP: Thrombotic thrombocytopenic purpura
CNIs: Calcineurin inhibitors
TPE: Therapeutic plasma exchange
ATN: Acute interstitial nephritis
